# Effects of Anesthesia and Species on the Uptake or Binding of Radioligands In Vivo in the Göttingen Minipig

**DOI:** 10.1155/2013/808713

**Published:** 2013-09-08

**Authors:** Aage K. O. Alstrup, Anne M. Landau, James E. Holden, Steen Jakobsen, Anna C. Schacht, Helene Audrain, Gregers Wegener, Axel K. Hansen, Albert Gjedde, Doris J. Doudet

**Affiliations:** ^1^Department of Nuclear Medicine and PET Center, Aarhus University Hospital, Nørrebrogade 44, Building 10G, 6th Floor, 8000 Aarhus, Denmark; ^2^Center of Functionally Integrative Neuroscience/MINDLab, Aarhus University, Nørrebrogade 44, Building 10G, 5th Floor, 8000 Aarhus, Denmark; ^3^Department of Medical Physics, University of Wisconsin, B1137 Wisconsin Institutes for Medical Research, UW-Medical Physics, Madison, WI 53706, USA; ^4^Translational Neuropsychiatry Unit, Aarhus University, Skovagervej 2, 8240 Risskov, Denmark and Pharmaceutical Research Center of Excellence, North-West University, Potchefstroom, South Africa; ^5^Department of Veterinary Disease Biology, Faculty of Life Sciences, University of Copenhagen, Thorvaldsensvej 57, 1871 Frederiksberg C, Denmark; ^6^Department of Neuroscience and Pharmacology, University of Copenhagen, Blegdamsvej 3B, 2200 Copenhagen N, Denmark; ^7^Department of Neurology, University of British Columbia, Purdy Pavillion, University of British Columbia, 2221 Wesbrook Mall, Vancouver, BC, Canada V6T 2B5

## Abstract

Progress in neuroscience research often involves animals, as no adequate alternatives exist to animal models of living systems. However, both the physiological characteristics of the species used and the effects of anesthesia raise questions of common concern. Here, we demonstrate the confounding influences of these effects on tracer binding in positron emission tomography (PET). We determined the effects of two routinely used anesthetics (isoflurane and propofol) on the binding of two tracers of monoamine function, [^11^C]SCH23390, a tracer of the dopamine D1 and D5 receptors, and the alpha2-adrenoceptor antagonist, [^11^C]yohimbine, in Göttingen minipigs. The kinetics of SCH23390 in the pigs differed from those of our earlier studies in primates. With two different graphical analyses of uptake of SCH23390, the initial clearance values of this tracer were higher with isoflurane than with propofol anesthesia, indicative of differences in blood flow, whereas no significant differences were observed for the volumes of distribution of yohimbine. The study underscores the importance of differences of anesthesia and species when the properties of radioligands are evaluated under different circumstances that may affect blood flow and tracer uptake. These differences must be considered in the choice of a particular animal species and mode of anesthesia for a particular application.

## 1. Introduction

Animals often serve as models of human diseases and pharmacological challenges. This practice includes the evaluation of the use of new radioligands for the study of conditions of relevance to the human population. The search for the most appropriate animal models is driven by multiple considerations of time, species, size, and cost of acquisition. For brain imaging such as positron emission tomography (PET), the size of the brain and the resolution of specific brain structures become important. Other factors must be considered when species, study design, and tracers are chosen for preclinical studies, or for the development of new tracers for human use, to the extent that they influence data analysis and interpretation. Among these factors, effects of anesthesia and specific physiological characteristics of the species can vary between humans and animals and therefore play a role. Despite the development of specific protocols and restraint devices, training in preparation of imaging of awake rodents and primates [1, 2] is time consuming; often only, few subjects prove to be suitable candidates for awake imaging [3], possibly biasing the population selection. Thus, most in vivo imaging depends on anesthesia to ensure immobility and reproducibility in long or multiple tracer imaging protocols.

The effects of individual anesthetics on the binding of radioligands and on their response to pharmacological challenges play a role in interpretation of the data [4, 5]. Similarly, different animal species may have specific known or unknown characteristics such as different liver enzymes, differential passage through the blood brain barrier (BBB), interactions between the tracer and the BBB ATP-binding cassette proteins (such as P-glycoprotein), receptor conformation, and polymorphisms, for example, that may affect interpretation and choice of tracer(s) for a given study.

Non-human primates such as macaques or baboons are the most useful and ideal animals for clinical applicability, but availability, cost, health status, and handling requirements associated with primate-specific biohazards, in addition to ethical issues, significantly reduce the feasibility of routine non-human primate use in PET studies in many countries. In recent years, the pig has become a model of interest for neuroimaging studies [6], with a gyrencephalic brain of a size similar to primates (75–90 grams in pigs versus 100–110 grams in macaques) and the opportunities for transgenic manipulations with less concern for availability, cost, and handling restrictions. The major issue for longitudinal studies in pigs is the rapid growth that precludes chronic studies. To circumvent this issue, easily trainable strains like the Göttingen minipig were specifically developed for research and long-term studies. It is thus of interest to carefully consider the use of swine for tracer development, kinetic analyses, and preclinical imaging.

While comparisons of the effects of anesthetized versus awake conditions in PET imaging have been published [2, 7], to the best of our knowledge, none have addressed the effects of various anesthetics on different tracers. In this study, we compared two commonly used, well-tolerated anesthetics, the inhalant isoflurane and the injectable propofol, on the binding of [^11^C]SCH23390, a well-validated and reversibly binding ligand of the dopamine (DA) striatal D1/5 receptors (with some affinity to the cortical 5HT2), and [^11^C]yohimbine, a new reversibly binding ligand of the alpha2 adrenoceptors, in Göttingen minipigs. Both anesthetics have attractive qualities for prolonged studies in swine. Propofol, a substituted phenol, is an ultrashort acting nonbarbiturate anesthetic which can be administered intravenously (IV) as a slow injection. It is cleared from plasma at a high rate, and consciousness returns more rapidly than with most other injectable anesthetics used in pigs. Propofol can also be administered by continuous infusion for prolonged anesthesia. It is metabolized primarily by conjugation in the liver. Isoflurane is an inhalation anesthetic with a broad safety margin, and the depth of anesthesia can be easily regulated. The recovery from isoflurane is also rapid due to its low solubility in blood, and metabolism in the liver occurs to a much lesser extent with isoflurane (0.2%) than with other inhalation anesthetics. In addition to their effects on tracer binding, we monitored their effects on the physiological variables of heart rate (HR), temperature, and O_2_ saturation.

## 2. Materials and Methods

### 2.1. Animals

The study followed a protocol approved by the Danish Animal Experimentation Inspectorate. Twelve adult Göttingen minipigs (females, 25–33 kg) from Ellegaard Minipigs ApS (Dalmose, Denmark) were fed a restricted pellet diet (DIA plus FI, DLG, Aarhus, Denmark) and fasted 16 hours prior to the study, with free access to tap water. The animals were housed at 20°C and 50–55% relative humidity with a photoperiod of 6:00 AM to 6:00 PM, and the air was changed 8 times per hour. The data were acquired as the baseline and control data for two separate studies performed on the same animals.

### 2.2. Anesthesia and Physiological Variables

We first premedicated the twelve minipigs with a mixture of 1.3 mg/kg midazolam (Midazolam “Hameln,” 50 mg/mL, Matrix Pharmaceuticals AS, Herlev, Denmark) and 6.3 mg/kg S-ketamine (S-Ketamine “Pfizer,” 25 mg/mL, Pfizer, Ballerup, Denmark) intramuscularly (IM). After placing an ear vein catheter (21G Venflon), we induced anesthesia with a mixture of approximately 1.3 mg/kg midazolam and 3.1 mg/kg S-ketamine IV. The minipigs were intubated, and anesthesia maintained at either 3.7–4.0 mg/kg/h propofol (propofol, 10 mg/mL, IV, B. Braun, Frederiksberg, Denmark) (*N* = 6) or 2.0–2.1% isoflurane (Forene, 100%, Abbott, Solna, Sweden) (*N* = 6). In both cases, we mechanically ventilated the minipigs with approximately 8 mL/kg/min O_2_ mixed 1 : 2.2 with medical air, as recommended for Göttingen minipigs [9]. The minipigs were placed on an electric blanket with thermostatic feedback to a rectal temperature monitor during imaging. We monitored and recorded body temperature (Temp, (°C)), heart rate (HR, (bpm)), and arterial oxygen saturation (SaO_2_, (%)) during the tomography for later analysis. Infusion of 0.5–1 liter saline continued for the duration of the study.

### 2.3. PET

We placed the minipigs in the high-resolution research tomograph (HRRT, CPS Innovation) in a dorsal recumbent position, with the head immobilized by a custom inflatable device to prevent motion. After a brief transmission scan, the minipigs were injected with [^11^C]SCH23390 and [^11^C]yohimbine at an interval of 2-3 hrs. To avoid possible bias and accommodate radiochemistry, the tracers were administered in a random order. In either case, we injected the tracer dose, adjusted to a standard volume of 10 mL with sterile saline IV, over 1 minute. Dynamic tomography began at injection and lasted 90 minutes in list mode. Postprocessing framing yielded 17 frames of increasing duration (0.5 to 15 min). Data were corrected for photo attenuation, scatter and random coincidences and reconstructed using a point spread function with 10 iterations and 16 subsets. The matrix dimensions were 256 × 256 × 207, and the voxel size was 1.2 mm^3^.  Table 1 shows the relevant variables, that is, animal's weight, injected dose, specific activity, and mass of injected compound.

### 2.4. Analyses and Statistics

PET images were coregistered to an atlas of an average magnetic resonance image (MRI) of 22 minipig brains [10], using Montreal Neurological Institute PET imaging software as previously described [11]. From the atlas, we segmented striatum, temporal cortex, frontal cortex, and thalamus as regions of interest. We used the cerebellum as reference region for the [^11^C]SCH23390 maps and a standard blood curve corrected for injected dose and weight as an input function for the [^11^C]yohimbine maps. The use of a population plasma activity curve for yohimbine data based on the absence of peripheral metabolism of yohimbine in swine [12] was previously validated using these 2 anesthetics during early tracer development measuring arterial input and protein binding. We did not find differences between input curves obtained under isoflurane or propofol anesthesia. The absence of peripheral metabolism may be due to either the lack of the necessary liver enzymes CYP2D6 and CYP2A4 or to some polymorphisms of the enzymes that preclude yohimbine recognition as a substrate. Time activity curves (TACs) were obtained from each region, and left and right hemispheres were averaged. For display and comparison with each other, the TACs from isoflurane and propofol anesthetized pigs were corrected for the amount of activity injected per kg of body weight to yield standard uptake values (SUVs). As previously described, the yohimbine data were analyzed with the plasma-input version of the Logan graphical analysis [13]. The last six frames (25 to 90 min) were used for the Logan method. The analysis yielded the total distribution volume *V*_*T*_.

For the SCH23390 data, we initially analyzed TACs with the Logan graphical analysis with cerebellum as the input function, yielding the distribution volume ratio (DVR) [14]. However, contrary to expectation and to the experience in humans and non-human primates, inspection of the TACs and the upward shift of the graphical representation of the Logan plot for SCH23390 in the striatum revealed a nearly irreversible uptake of the tracer. A universal graphical method (UGA) is the ideal analysis method under these circumstances as it allows the estimation of both the uptake rate constant Ki and the equilibrium distribution volume [15–17]. The method is essentially a Gjedde-Patlak graphical analysis modified for use with a reference tissue rather than an input function [18, 19], for tracers that are mostly irreversibly binding, but also include a slow efflux of the tracer to plasma as a first-order process with the rate constant *k*_loss_. The ratio (Ki/*k*_loss_ + intercept) is proportional to an equilibrium distribution volume of the most slowly clearing kinetic component. With a reference tissue TAC as input function, the method yields the normalized uptake rate constant Ki* and the target-reference equilibrium distribution volume ratio DVR (Ki*/*k*_loss_). 

The fitted frame ranges varied according to conditions. Thus, all but the first three minutes of the study were included in the regression for the UGA graphical analysis, while the yohimbine regression included only the last six frames, representing the 25–90-minute period of the tomography. We did analysis of variance (ANOVA) with Bonferroni correction for multiple comparisons in order to assess statistical significance between the two anesthetics.

## 3. Results

### 3.1. Physiological Variables

The values of physiological variables differed very little between the two anesthetics as listed in Table 2 for average values of heart rate, oxygen saturation (SaO_2_), and body temperature. The reference value for heart rate in Göttingen minipigs is 68–98/min [9]. The heart rate was higher for isoflurane-anesthetized minipigs (mean 113/min) than for propofol-anesthetized minipigs (mean 76/min) throughout the course of the study (*P* = 0.0078). The mean SaO_2_ was 98% for both groups, consistent with the reference interval for Göttingen minipigs [9]. The mean body temperatures (isoflurane: 36.7 C and propofol: 36.3 C) remain close to the standard body temperature of 37-38°C in minipigs [9]. Aside from the heart rate, overall differences of physiological variables between the two anesthetic groups of animals were small, and the animals had uneventful recoveries from the anesthesia.

### 3.2. Tomography

**Figure 1:**
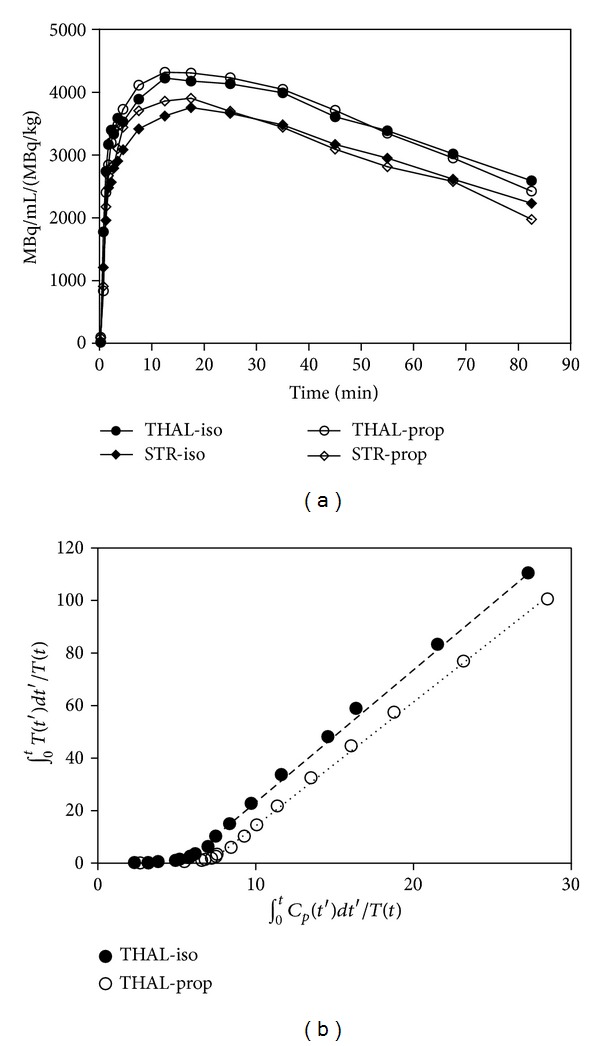
Time activity curves (TACs) and Logan plots for yohimbine. (a) Averaged yohimbine TACs for a region of high binding (thalamus) and a region of lower binding (striatum) in pigs (data corrected for amount of injected activity per kg body weight) for comparison of isoflurane and propofol anesthesia. (b) Logan plots of yohimbine thalamus data for 2 representative pigs, one anesthetized with isoflurane (filled circles), and one anesthetized with propofol (open circles). Note the straightness of the fitted line.

The choice of anesthetic had no effect on the estimate of any of the kinetic parameters determined from the yohimbine data. The TACs shown in Figure 1(a) are the average of the 6 animals of each study, normalized to amount of activity injected per kg of body weight in order to permit comparison of the groups.  Figure 1(b) shows Logan plots of the thalamus with corrected standard arterial plasma curve as the input function for representative animals anesthetized with isoflurane and propofol. Note the linear regressions that yield the total steady-state distribution volumes *V*_*T*_. There was no significant difference of the *V*_*T*_ estimates for any region considered with the two anesthetics (Table 3).

**Figure 2:**
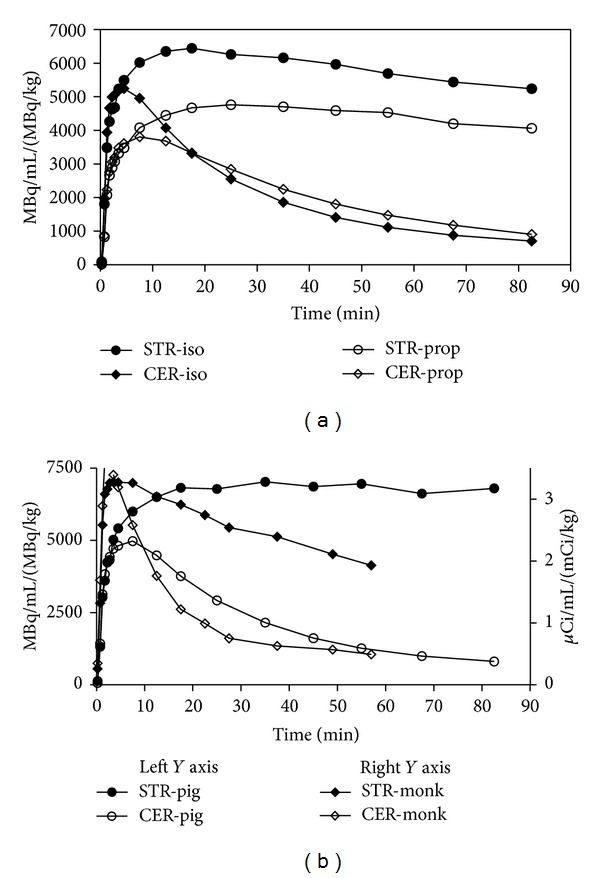
Time Activity Curves (TACs) for SCH23390. (a) SCH23390 TACs (corrected for amount of injected activity per kg of body weight) for striatum and cerebellum in isoflurane- and propofol-anesthetized pigs. (b) For comparison, TACs (corrected for injected activity per kg of body weight) in a representative isoflurane-anesthetized pig and a representative isoflurane-anesthetized monkey (data directly taken from an earlier published study [8]).

In contrast, the binding of tracer SCH23390 yielded surprising differences, with respect to both the two anesthetics and to the kinetics of tracer uptake into pig brain compared to evidence from human and non-human primate brains [8, 20]. We initially compared the binding of [^11^C]SCH23390 in minipigs receiving isoflurane or propofol for the duration of the tomographies, using the simplified reference tissue method (SRTM) [21] and the Logan graphical analysis [14] with cerebellum activity as the input function, methods of choice in many human and non-human primate studies [8]. Inspection of the TACs (normalized to the amount of activity injected/kg body weight for easy comparison of the two groups) showed higher uptake in animals maintained on isoflurane anesthesia than in animals treated with propofol, suggesting reduced perfusion.  Figure 2(a) shows the shape of the averaged TACs for the striatum and cerebellum in propofol (open symbols) and isoflurane (closed symbols) anesthesia, which were notably different from the time course expected from previous reports cited above. For comparison, Figure 2(b) shows SCH23390 TACs in one representative isoflurane-anesthetized pig (circles) and a representative isoflurane-anesthetized monkey (diamonds) during the 60 minutes after injection of a dose of SCH23390 adjusted to 10 mL and injected over 1 minute as in pigs. The activity cleared much more slowly from the pig striatum during the 90 minutes of the study than from primate striatum during 60 minutes.

**Figure 3:**
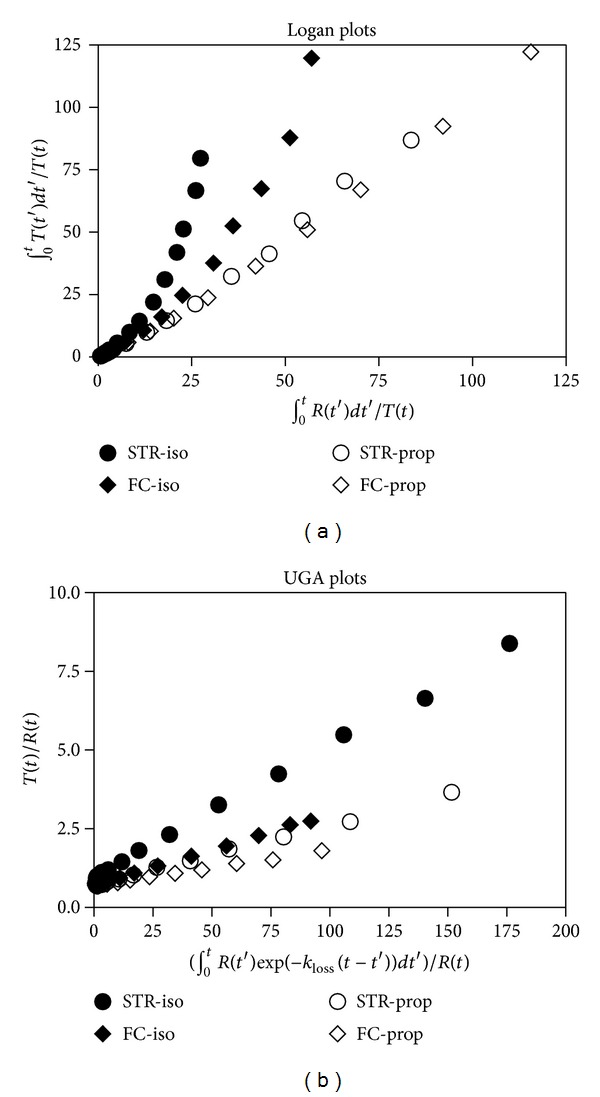
Logan and UGA plots for SCH23390. (a) Logan plots for the striatum (STR) and frontal cortex (FC) of SCH23390 data in representative pigs anesthetized with isoflurane (iso) or propofol (prop). Note the upward curvature characteristic of an irreversible tracer. (b) UGA plots of the same data.

Consistent with the behavior of the TACs, the Logan plots of tracer SCH23390 uptake in both isoflurane and propofol anesthesia did not yield the linear regression characteristic of reversible binding but rather the upwards curvature characteristic of irreversible or slowly reversible accumulation, as shown in the representative Logan plots of Figure 3(a). Therefore, we reprocessed the data with a graphical analysis that takes slow efflux into account, as shown in Figure 3(b) of the same animal shown in Figure 3(a) for the striatum (STR), an area of high DAD1 receptor density, and frontal cortex (FC), an area of low DAD1 receptor density. The rate constant *k*_loss_ assumes the value that best causes the resulting points to fit a linear regression, and the estimate of the rate constant Ki* is the slope of the linear regression.

In the frontal cortex, propofol caused a decline of the estimate of slope as well as of the estimate of *k*_loss_. In the striatum, however, propofol caused a very similar reduction of slope, but without the corresponding reduction of *k*_loss_. While detailed analysis of the kinetics of SCH23390 is not possible at this time, we mention these observations from pigs and primates as an example of species-specific differences. Regardless of the analysis, there are no significant differences in the Ki*/*k*_loss_ of SCH23390 binding in the isoflurane and propofol-anesthetized animals for the region of low receptor density such as cortex or thalamus, but there was a clear significant difference in the region of high receptor density, striatum (Table 4).

## 4. Discussion

From the results, we conclude that differences in response of physiological variables to two anesthetics of dissimilar modes of action, one inhalant and one injectable, do not account for differences of radioligand binding to neuroreceptors in the central nervous system. Indeed, only minor differences of the physiological variables were noted in the pigs anesthetized with isoflurane and propofol. We found no differences of SaO_2_ or body temperature between the propofol and isoflurane groups. Not unexpectedly, the heart rate was slightly lower in the propofol group than in the isoflurane group. In both groups of minipigs, the heart rate was low at the beginning and increased by 32–36% over time. A reason for the lower heart rate at the beginning of the anesthesia could be an effect of the combination of ketamine and midazolam used for premedication and induction of anesthesia. However, the tomographies occurred at least 2-3 hours after initial premedication, and thus the role of premedication may be insignificant.

Lower heart rate in the beginning of anesthesia is a common observation in most animal studies with most anesthetics. The increase in heart rate during propofol or isoflurane sedation is in agreement with human and animal studies [22] in which a higher heart rate usually is observed with isoflurane. It would have been of interest to measure arterial pressure throughout the study; however, because these animals were involved in a series of long-term, longitudinal studies and multiple tomographies, we reduced the burden placed on them by not placing an arterial line. As the animals were artificially ventilated, respiratory rate was constant between anesthetics.

An observation from this study is the suggestion of species differences influencing tracer binding. While the study was not designed to investigate species differences, comparison between our pig data and the literature in other species, even using well-validated and extensively used tracers, was striking. Both SCH23390 and yohimbine provided clear examples. Yohimbine is a useful tracer for swine as it has little or no peripheral metabolism, allowing for the use of a substitute population average of the arterial plasma activity to estimate the total distribution volume. In human and non-human primates, the drug is variably metabolized [23], and quantification of tracer kinetics in primates will require acquisition of individual arterial input functions and metabolite correction.

Similarly, the kinetic behavior of SCH23390, a widely and routinely used tracer of the DAD1/5 receptors in primates, appears to be highly species dependent and to require different kinetics of quantification, due to reversible binding in primates and irreversible binding in pigs during the time of a PET session. The reasons for the different kinetics of SCH23390 binding in primates and pigs, irrespective of anesthetic, are unclear. We hypothesize that differences of peripheral metabolism, including possible lack of peripheral metabolism altogether, as well as possible differences of blood-brain barrier transport, may play a part in the retention and apparent irreversibility of SCH23390 or SCH23390 metabolite binding in the brain. Because of the irreversibility of the uptake, we determined the kinetics of SCH23390 binding in the pig brains by graphical analysis of the uptake of irreversibly or slowly clearing tracers [15, 16, 18, 19].

Previous studies of other tracers also demonstrated that species differences, for example, the poor [^18^F]fluorodopa uptake in rats in the absence of inhibition of catechol-O-methyltransferase, compared to human and non-human primates, are well described [24, 25]. On the other hand, the NK1 antagonist [^18^F]SPA-RQ defluorinates extensively in non-human primates, leading to significant accumulation of activity in bone, but not in human subjects, making it a better tracer for human studies than predicted from non-human primates [26]. Similarly, discrepant kinetic findings between primates and pigs have already been reported, for example, N-methylspiperone [27]. These discrepancies recently led to many new tracers being pushed to rapid “first in human” studies using microdosing paradigms for initial evaluation before full validation in animal studies [28, 29].

A notable result of the study is the variable effect of anesthesia on different tracers, clearly demonstrating the impact of individual tracers' kinetics on binding characteristics; there is no anesthesia-induced effect on the binding of yohimbine to the alpha2 adrenoceptors. With its rapidly reversible binding, the yohimbine accumulation is insensitive to the lowering of brain perfusion by propofol, and brain uptake appears to be limited by receptor density and affinity rather than by flow. 

However, we found striking differences of SCH23390 binding between the two anesthetics. The lower SCH23390 binding in animals anesthetized with propofol likely can be attributed to a decrease of cerebral blood flow (CBF). In rats, chloral hydrate and ketamine anesthesia, which increase CBF, increased the striatal binding of SCH23390 by 36% and 46%, respectively, compared to nonanesthetized rats, while the CBF depressor pentobarbital decreased the binding by 41% [30]. Several studies have demonstrated that propofol decreases CBF and the cerebral metabolic rate of oxygen [31, 32], while isoflurane increases CBF [33, 34]. In pigs, propofol has been shown to cause a 35% reduction of the cerebral metabolic rate of oxygen and a 39% decrease of CBF, measured as cerebral venous outflow [35]. In our own pig studies, the reduced perfusion due to propofol anesthesia is clearly seen not only in the striatum but also in the cerebellum (Figure 2(a)), a region devoid of specific binding, especially in the first 10–15 min of the cerebellum time activity curves. In regions of low D1 binding, Ki* and *k*_loss_ are equally affected by blood flow (30–40% decrease; see Table 4), and the Ki*/*k*_loss_ ratio is not significantly different between anesthetics. The finding of a 42% decrease of the striatal Ki*/*k*_loss_ ratio of SCH23390 in propofol-anesthetized pigs compared to isoflurane-anesthetized pigs, on the other hand, is likely due to differential binding characteristics possibly associated with the presence of brain metabolites. The slow, nearly irreversible binding of SCH23390 explains the significant effect in regions of high receptor density (striatum) but not in regions of low receptor density. The low reversibility of SCH23390 binding, although exaggerated in the pig compared to primates, is not a completely unexpected finding. Schultz et al. already reported the persistence of SCH23390 in rat striatum up to 3 hrs after injection, despite rapid plasma clearance [36]. Gifford et al. hypothesized that the diffusion of SCH23390 out of brain is hindered by a binding-rebinding process of the tracer to neighboring receptors, an effect which is magnified in regions of very high receptor density (high binding potential) [37]. Thus, the interaction of the depressing effect of propofol on CBF combined with SCH23390's nearly irreversible kinetics leads to (1) significantly lower receptor binding in high receptor density regions for propofol compared to isoflurane anesthesia and (2) significant differences in the tracer influx in all regions, regardless of receptor density (Table 4). The original study was not designed to specifically address effects of blood flow, and we did not measure it, and thus we cannot exclude an effect of flow or change in pCO_2_ in our data. However, the studies with yohimbine and SCH23390 were conducted similarly in random order, and we did not detect effects attributable to flow in any region of the yohimbine studies suggesting that large perfusion differences may be accountable for the SCH23390 binding alone.

We do not discount the possibility of other effects of anesthesia. While the influence of propofol appears more pronounced on SCH23390 binding, it is likely that both anesthetics exert some subtle effect on the binding of both tracers compared to the awake condition. Propofol, at sedative doses, reduces cerebral glucose metabolism in cortical regions and during the unconscious state, decreases glucose metabolism in subcortical regions, especially in the hippocampus and thalamus [38]. A study of the uptake of [^18^F]FDG into brain in mice in awake and anesthetized states found that both isoflurane and a ketamine/xylazine mixture decreased the uptake compared to awake mice [39].

Anesthesia-related effects can be avoided by studies in awake animals, but this is problematic from the animal welfare point of view as head fixation or restraint induces stress, which can greatly influence the results of brain studies. Particularly, handling and restraint of awake rats have been shown to influence the DA system and affect the binding potential of PET imaging tracers [7, 40]. In awake animals, extensive training over several days can mitigate some effects of stress, and measurements of the HPA axis parameters, including prolactin, can provide an estimate of stress levels. Extensive and time consuming training and often specifically designed or adapted restraints and/or scanning devices are necessary to perform awake imaging in large animals such as non-human primates or pigs. Furthermore, not all animals are amenable to such training, raising the issue of possible population bias [3]. This study however only aimed to compare the respective effects of 2 common anesthetics on 2 tracers with different kinetics.

## 5. Conclusions

In this paper, we demonstrate that the choice of anesthetics and their variable effects on perfusion play a significant role in the analysis of some tracer kinetics and interpretation of their data while other tracers, which are more insensitive to flow changes, show little differences in binding characteristics. Furthermore, we show that physiological differences between species may affect tracer binding in often poorly appreciated ways, even when using well-validated tracers, bringing to the forefront the need to carefully consider conclusions from neuroimaging studies in animals, not only for tracer or drug development, but also for evaluation and validation of animal models of diseases and new therapies.

## Figures and Tables

**Table 1:** Scan parameters shown as mean ± SD and significance.

	Weight (kg)	[^11^C]SCH23390			[^11^C]Yohimbine		
		Inj. Dose (MBq)	SA (GBq/*µ*mol)	Mass (*µ*g)	Inj. Dose (MBq)	SA (GBq/*µ*mol)	Mass (*µ*g)
Propofol	29 ± 4	229 ± 88	55 ± 34	1.81 ± 1.8	333 ± 49	105 ± 66	1.59 ± 1.0
Isoflurane	27 ± 2	244 ± 47	89 ± 106	1.67 ± 1.4	302 ± 18	219 ± 307	1.22 ± 1.0
*P* value	N.S.	N.S.	N.S.	N.S.	N.S.	N.S.	N.S.

**Table 2:** Physiological parameters measured with both anesthetics. Data are shown as mean and range.

	HR (beats/min)	SaO_2_ (%)	Temperature (°C)
Propofol	76 (49–99)	98 (96–99)	36.3 (35.9–36.7)
Isoflurane	113 (86–143)	98 (97–99)	36.7 (35.5–38.0)
*P* value	<0.01	N.S.	N.S.

HR: heart rate. SaO_2_: oxygen saturation.

**Table 3:** Logan *V*_*T*_ values (mL/g) (mean ± SD) for yohimbine studies (*N* = 6) using the two anesthetic compounds.

Anesthetic	Thalamus	Striatum	Frontal *C*_x_	Temporal *C*_x_	Cerebellum
Isoflurane	4.99 ± 0.51	4.29 ± 0.55	4.38 ± 0.71	4.29 ± 0.37	4.19 ± 0.72
Propofol	5.49 ± 1.17	4.60 ± 1.16	4.89 ± 1.16	4.85 ± 1.16	4.20 ± 1.04

**Table 4:** Kinetic parameter values (mean ± SD) from the UGA analysis for SCH23390 studies (*N* = 6) using the two anesthetic compounds for the average of 3 nonstriatal regions (thalamus, frontal, and temporal cortices) and striatum.

	Ki* (1/min)	*k*_ loss_ (1/min)	Ki*/*k*_loss_
Non striatal regions			
Isoflurane	0.0268 ± 0.008	0.032 ± 0.011	0.84
Propofol	0.0166 ± 0.007*	0.023 ± 0.010	0.72

Striatum			
Isoflurane	0.0465 ± 0.018	0.009 ± 0.004	5.16
Propofol	0.0298 ± 0.008*	0.010 ± 0.003	2.98*

*P < 0.05.
